# Chemical profiling and anti-psoriatic activity of marine sponge (*Dysidea avara*) in induced imiquimod-psoriasis-skin model

**DOI:** 10.1371/journal.pone.0241582

**Published:** 2020-11-30

**Authors:** Mostafa Khaledi, Behzad Sharif Makhmal Zadeh, Annahita Rezaie, Melika Nazemi, Mehdi Safdarian, Mohammad Bagher Nabavi

**Affiliations:** 1 Marine Pharmaceutical Science Research Center, Department of Pharmacognosy, Ahvaz Jundishapur University of Medical Sciences, Ahvaz, Iran; 2 Nanotechnology Research Centre, Ahvaz Jundishapur University of Medical Sciences, Ahvaz, Iran; 3 Department of Pathobiology Faculty of Veterinary Medicine, Shahid Chamran University of Ahvaz, Ahvaz, Iran; 4 Gulf and Oman Sea Ecological Research Center, Iranian Fisheries Science Research Institute (IFSRI), Agricultural Research, Education and Extension Organization (AREEO), Bandar Abbas, Iran; 5 Halal Research Center of Islamic Republic of Iran, Tehran, Iran; University of British Columbia, CANADA

## Abstract

Since Marine sponge *Dysidea avara* is regarded as a source of anti-inflammatory compounds, we decided to evaluate its potential anti-psoriatic activity in a psoriasis Imiquimod-induced in the mouse model. Psoriatic mice were treated with three different methanolic extracts of *Dysidea avara* compared with betamethasone-treated mice in in- vivo studies. Clinical skin severity was assessed with the psoriasis area index (PASI), whilst ELISA detected the expression of TNF-α, IL-17A, and IL-22. *Dysidea avara* activity was studied by employing GC-MS (to distinguish compounds), HPTLC (for skin permeation and accumulation), and SEA DOCK to predict single compound potential anti-inflammatory activity. After 7 days of treatment, mice treated with *Dysidea avara* displayed a dose-dependent, statistically significant improvement compared to controls (*p*< 0.001). In line with the clinical results, ELISA revealed a statistically significant decrease in IL-22, IL-17A, and TNF-α after treatment; the same SEA DOCK analysis suggests a possible anti-psoriatic activity of the extracts.

## 1. Introduction

Psoriasis was initially regarded as a main epidermal proliferation disorder that causes excessive keratinocyte proliferation in the epidermis. Although plaque psoriasis is traditionally a dermatological disease, due to its erythematous, desquamative, and infiltrate lesions [1–3], nowadays this tendency is inverting, highlighting the systemic inflammatory behaviors of psoriasis [4]. This new vision is empowered by the growing numbers of comorbidities [5], and by findings that psoriasis-related system inflammation may also affect other organs [6], providing the rationale for supposed pathogenesis. This hypothesis was further tested by treating psoriasis, and analysis of the inflammation in other organs, finding both in the mouse model [7] and humans [8] that cutaneous manifestations of psoriasis deeply influence and even provoke related co-morbidities. Although several pathogenic models of psoriasis were suggested, they are still unable to capture the complexity of psoriatic disease [1], a disease that shares a genetic component that may display the pathological phenotype upon environmental triggers. From a therapeutical point of view, psoriasis is a chronic disease with a relapsing-remitting behavior; thus, patients undergo treatments for a long time and have a high rate of resistance [9], leading clinicians to develop switching/combination strategies, new delivery approaches and also new molecules. Although several biologics are available, anti-psoriatic drugs may also be enriched by natural extracts, that, after proper study, may enter the pharmacopeia, as in the case of fumigates [10]. These marine natural products exhibit a broad variety of biological activities that play a significant role in drug discovery and development for human disease therapy [11]. Marine bioactive compounds generally have a distinctive chemical structure and elevated biological activity due to the features of marine settings [12]. New structures and metabolic pathways encourage chemistry researchers and the biological activities of these compounds are highly appealing to drug researchers [13]. Sponges demonstrated a high ability in chemical defense and a resistance to natural conditions. Marine sponges are a good source of metabolites that are essential for their chemical defense against predators, spatial rivals, and corruption. Many studies are attempting to discover new anti-inflammatory molecules and marine sponges are a wealthy source of pharmaceutical-used, active biological compounds [14]. A broad variety of operations are reported to involve bioactive natural compounds extracted from the marine sponge, including anti-inflammatory, antioxidants, and other agents [15, 16]. Marine sponges have offered numerous new secondary metabolites with diverse chemical structures and powerful anti-inflammatory activity [16]. Different marine metabolites, with different modes of action and activity levels, have been introduced [17, 18].

This study was conducted to classify the different chemical compounds and determine the efficacy, of *Dysidea avara* methanolic extract (grown in the Persian Gulf, Iran) in psoriasis caused by Imiquimod (IMQ) in the mouse model. We used a mouse model to replicate psoriasis elements. IMQ is an immune activator, effective in inducing the rapid psoriasis of desquamation, erythema, and thickening effects on mice and human skin. This model demonstrates some parts of the properties in psoriasis that are based on the IL-23, IL-22, and IL-17A. Recently, psoriasis-like dermatitis has been revealed by the topical application of Imiquimod, a toll-like receptor (TLR) ligand 7 and 8 [19].

## 2. Materials and methods

### 2.1. Preparations of the marine sponge extract

The marine sponge of *Dysidea avara* was collected by hand using SCUBA at the depths of 15–30 m from the Persian Gulf (Hengam Island, Iran) (N 26°36’43”and N 26°41’15”, E 55°54’40”and E 55°54’55”) on August 2018. The species was identified by a taxonomist at the Persian Gulf and Oman Sea Ecological Research Organization (Bandar Abbas, Iran). The sponge was transferred to the lab in an icebox, washed with running tap water and distilled water. The marine sponge was cut into tiny pieces (1 cm) and, after drying by freeze dryer, maintained at −24°C until extraction. A total of 100 g of dried powder was weighed and extracted with methanol (1000 cc) at room temperature for 72 h in three cycles. The extract was filtered and evaporated in a rotary vacuum evaporator (40°C) to remove methanol. Then, the residue was dried by freeze dryer.

### 2.2. Animal housing

Female BALB/C mice (8 to 11 weeks old) were purchased from the Pasteur Institute of Iran. All animal procedures have been approved by the Animal Ethical Committee, Ahvaz Jundishapur University of Medical Sciences, Ahvaz, Iran (No: IR.AJUMS.ABHC.1397.084) following animal use and care guidelines. All surgery was performed under sodium pentobarbital anesthesia, and all efforts were made to minimize suffering. The animals were adapted to laboratory conditions by the initiation of the experiment. They were given feed in the form of pellets and water adlibitum. Thirty-six mice were randomized according to their body weight and divided into six groups (G1–G6), comprising of six mice each with a mean body weight variation not exceeding ± 20% between the groups. The animals were shaved back(nearly 2 × 3 cm^2^) and given 2 days of acclimatization [20].

### 2.3. Psoriasis-like skin model

A total of 50 mg IMQ (Glen mark Pharmaceuticals, Mumbai, India) was applied on the shaved backs of mice for 11 consecutive days in Group 2–6 (G2–G6), and Group 1(Control) received 500 μL of distilled water on each mouse’s shaved dorsal surface as control. The daily application of IMQ on mouse back skin induced epidermal proliferation, abnormal differentiation, neo-angiogenesis and epidermal expression of IL-23, and IL-17A, TNF-α [21]. The severity of inflammation was evaluated daily by standard psoriasis area index (PASI) [21]. Based on PASI score, erythema, scaling and thickening were scored independently every 24 h based on the scale 0 to 4: 0 = none; 1 = slight; 2 = moderate; 3 = marked; 4 = very marked. Skin caliper measurements are performed every day using a digitrix II micrometer. Pictures of lesions before daily treatment were taken and used for scoring redness by a dermatologist. The severity of inflammation was assessed from 0 to 4 by a pathologist through determining acanthosis intensity, parakeratosis, thickening of the epidermis, and inflammatory cell infiltration. Total scoring of erythema, redness, and thickness was calculated and used for the evaluation of topical treatment effectiveness.

### 2.4. Treatment of psoriasis

After 11 days of IMQ application, all the animals were re-randomized based on mean PASI score and subjected to six groups. The application of IMQ (50 mg) has been continued across the treatment phase in G2–G6 groups, to ensure that recovery, if any, in the mice is due to the extract and not because of self-healing. G1 group did not receive IMQ and was considered as normal skin. Animals in the G2–G4 (L, M, H) groups were treated with 500 μL of three different concentrations (200, 400, and 600 mg/kg/day) of methanolic extract dissolved in PBS containing % 0.2 w/v of surfactant (20% span 20, 80% tween 80), Group 5 (betamethasone) received Betamethasone ointment (BETAMETHASONE-NAJO 0.1% 15 GR Ointment^®^) daily on to dorsal skin that is comparable concentration used in psoriasis treatment, and Group 6 (IMQ) was treated with 500 μL vehicle solvent. G1, without the disease, was treated with Distilled water (500 μL). The success of treatment was evaluated by PASI score, histopathological evaluation, and levels of TNF-α, IL-17A, and IL-22 [21].

### 2.5. Histological studies

At the end of the experiment, all inflamed mice were sacrificed using ketamine/xylazine, and then skin samples were removed and fixed with 10% formaldehyde, embedded into the paraffin and cut to a thickness of 5 μm by a microtome (Leica RM 2245. Buffalo, NY, USA). The samples were stained with hematoxylin and eosin, and photographs were prepared [22].

### 2.6. Enzyme-linked immunosorbent assay of TNF-α, IL-17A, and IL-22 in skin

The tissue samples (700 mg) were used for the quantification of TNF-α, IL-17A, and IL-22 in all the groups by ELISA. After 14 days of treatment, the skin of each group was collected and immediately frozen in liquid nitrogen and stored at −80°C. The samples in the RIPA lysis buffer were suspended and homogenized by the probe type sonicator for 10 s. The lysed skin back tissue was centrifuged for 10 minutes at 12,000 RPM, and the supernatant was collected and the cytokine concentration was quantified using the manufacturing protocol for ELISA kit (LEGEND MAX, San Diego, CA, USA). The experiment was carried out in triplicates, calculating the means and standard deviations [22].

### 2.7. GC-MS analysis

The marine sponge extract was subjected to derivatization before GC-MS analysis [31]. 1 mg of the methanolic extract was added in Dichloromethane (100 μL), vortexed, and dried under the nitrogen gas. The residue was mixed with 50 μL N-methyl-N-trimethylsilyl-trifluoroacetamide (MSTFA), heated at 80°C for 15 min, cooled and stored at −20°C until GC-MS analysis [21, 23].

An Agilent 6890 gas chromatograph (Agilent Technologies, Palo Alto, CA, USA) equipped with a selective mass detector of 5973N and an Agilent 19091S-413HP-5MS 5% Phenyl Methyl Silox capillary column (30 m/320 μm/0.25 μm) was used to perform the GC-MS analysis. One μL of the silylated extract was injected into the GC-MS with a split injection mode of 20:1 split ratio. The program of oven heating was: 80 to 100°C (rate 20°C/min, hold 1.0 min), 100 to 150°C (rate 10°C/min, hold 1.0 min), 150 to 180°C (rate 15°C/min, hold 1.0 min), 180 to 200°C (rate 10°C/min, hold 1.0 min), followed by an increase to 280°C (rate 10°C/min, hold 5 min). The injector temperature was 260°C and Helium gas was used as a carrier in a steady flow mode at a flow rate of 1 mL/min. Inlet and GC/MS interface temperatures were, respectively, kept at 250°C and 280°C. EI sources and the quadruple analyzer was kept respectively at 230°C (250°C maximum) and 150°C (200°C maximum). MS scanning in full scanning mode ranged from small to high mass (50 M/z–700 M/z). NIST2008 Program was used for matching characterized compounds.

### 2.8. The prediction of active compounds

The SEA SEARCH SERVER estimated the possible anti-inflammatory targets of all the compounds determined by GC-MS [22].

### 2.9. *In vitro* permeation studies

In vitro permeation study was performed based on cholan-24-oic acid, 3, 12-dioxo-, (5.beta.) (Cholan), which is one of the major compounds found in the marine sponge. Therefore, it was considered as a biomarker in permeation studies through normal and psoriatic skin by the Franz diffusion cells [24].

The skin was put between donor and receptor compartments that faced the donor with the skin. The receptor is PBS buffer containing % 0.2 w/v of surfactant (20% span 20, 80% tween 80), (pH 7.4). The donor (5 mL) was loaded with methanolic extract (8 mg/mL).

For permeants, the active diffusion area was 0.785 cm^2^. The receptor temperature was regulated at 37°C, and the receptor stirred at 100 RPM. A 2 ml receptor phase solution aliquot was removed at 0.5, 2, 4, 6, 8, 24, and 48 h and replaced by the fresh buffer including surfactants. All samples of psoriasis skin and normal skin were analyzed by the high-performance thin-layer chromatography (HPTLC, CAMAG, Muttenz, Switzerland) for estimating cholan concentration in skin and receptor [24, 25].

### 2.10. HPTLC method

#### 2.10.1. Chromatographic conditions

Plates were developed using a mobile phase consisting of toluene: hexane: methanol: ethyl acetate: acetic acid (60:15:17.5:5:2.5). Linear ascending development was carried out in a 7 cm twin trough glass chamber equilibrated with the mobile phase. The optimized saturation time of the mobile phase chamber was 30 minutes at 25°C. Twenty-five milliliters of the mobile phase was used for each development (10 mL in a trough containing the plate and 15 mL in other troughs) to migrate a distance of 70 mm, which took 28 minutes. Following the development, the TLC plates were thoughtfully dried [26].

#### 2.10.2. Mobile phase and migration

Densitometry scanning was conducted in observance mode on a Camag TLC scanner III and operated by planar chromatography version 1.4.4 of winCATS. The spots were analyzed at 254, 366, 525, and 200–700 nm wavelengths. The length and width of 4 and 0.2 mm were the slit sizes, with a scanning speed of 20 mm/s. This includes 70%–90% of the length of the application band, which was 6 mm in this case. The concentration of a cholan was determined from the intensity of diffusely reflected light and evaluated as peak areas against concentrations using a linear regression equation [27].

#### 2.10.3. Densitometric analysis

Densitometric scanning was conducted in an observance mode on Camag TLC scanner III and operated by planar chromatography version 1.3.4 of winCATS. A deuterium lamp was the source of radiation. The spots were analyzed at 254, 366, and 200–700 nm wavelengths. The length and width of 5 mm and 0.45 mm with a scanning speed of 20 mm/s were the slit sizes used in this study. This includes 70%–90% of the length of the application band, which was 6 mm in this case. The monochromatic bandwidth was set at 20 nm. The concentration of a cholan was determined from the intensity of diffusely reflected light and evaluated as peak areas against concentrations using a linear regression equation [28].

#### 2.10.4. Method validation

The working range was evaluated against the concentration of standards in the g/band by plotting chromatographies peak areas. The squared regression analysis in Excel was used to establish linear ranges. Specificity was evaluated by the capacity to separate samples from the optimized mobile phase. Repeatability was evaluated by applying three repetitions of each standard within the calibration curve at six concentrations (20, 50, 100, 200, and 400 μg/ml). As a relative normal deviation (percent RSD), the variance between repetitions was demonstrated. Measurement sensitivity was estimated in terms of the limit of quantification (LOQ) and a limit of detection (LOD). LOQ and LOD have been calculated using equation LOD = 3 Sd/B and LOQ = 10 Sd/B, where Sd is the standard deviation of the normal peak fields (*n* = 3), taken as a noise measure, and B is the slope of the respective calibration curve [29].

### 2.11. Statistical analysis

The data are shown as means ± SD. ANOVA produced statistical comparisons on a single-way basis. The use of GraphPad Prism 5.0 was deemed statistically relevant at *p*< 0.05.

## 3. Results

### 3.1. PASI scoring analysis

After 11 days of topical application of IMQ, psoriasis plaques were induced on the mice’s dorsal portion. IMQ-induced dermatitis in mice strongly resembles psoriatic human plaque lesions in morphological and histopathological characteristics and is ideal for rapid in vivo testing of anti-psoriasis drugs [30].

The PASI score is a quantitative index that reflexes the severity of the psoriasis lesions. The effect of different treatments on the PASI score is shown in Table 1. Based on these results, the PASI score change in the negative control group (without any treatment after psoriasis induction by IMQ) was only 6.4%, which indicates no self-healing of the lesions. Among treated groups, 600 mg/kg/day extract of *Dysidea avara* demonstrated the highest reduction in PASI value, followed by 400 and 200 mg/kg/day extract. All *Dysidea avara* extracts reduced PASI values significantly more than betamethasone. Therefore, *Dysidea avara* indicated an effective healing effect on plaques of psoriasis, in that its effect showed a dose-response effect.

**Table 1:** The change in PASI scores during the treatment period (Mean ± SD, *n* = 5).

Treatments	PASI		% decrease in PASI
	Induction of psoriasis	End of therapy	
IMQ-cream	8.79 ± 0.63	8.22 ± 0.57	6.4
Aqueous treated	8.44 ± 0.71	7.39 ± 0.52	7.6
600 mg/kg/day	8.52 ± 0.59	0.49 ± 0.11	96.6*
400 mg/kg/day	8.65 ± 0.79	0.77 ± 0.1	91.1*
200 mg/kg/day	8.57 ± 0.50	0.93 ± 0.12	89.2*
BMT	8.48 ± 0.69	2.83 ± 0.05	67*

* indicates a significant difference in PASI score between before and after treatment

PASI: psoriasis area index

### 3.2. Histopathological observations

**Fig 1:**
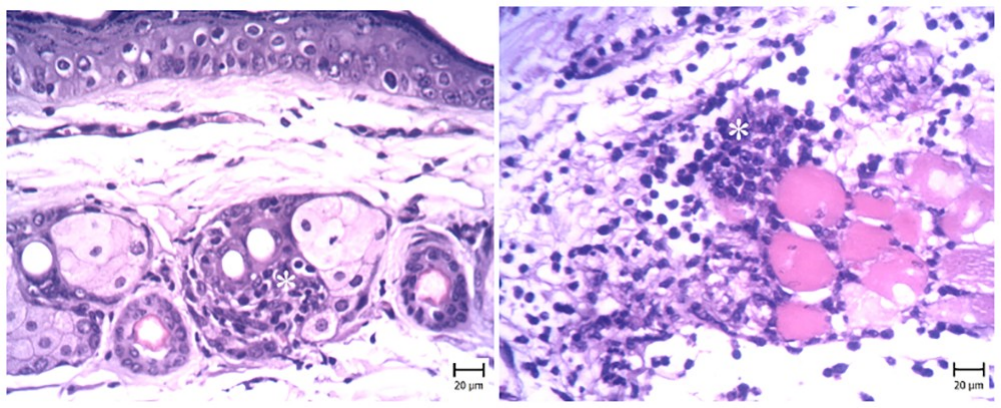
Skin. Mouse in the G6 group (IMQ) (H&E). (Bar = 20 μm). Note the infiltration of inflammatory cells (white asterisks) in the dermis around sweat glands and between connective tissue and muscles.

**Fig 2:**
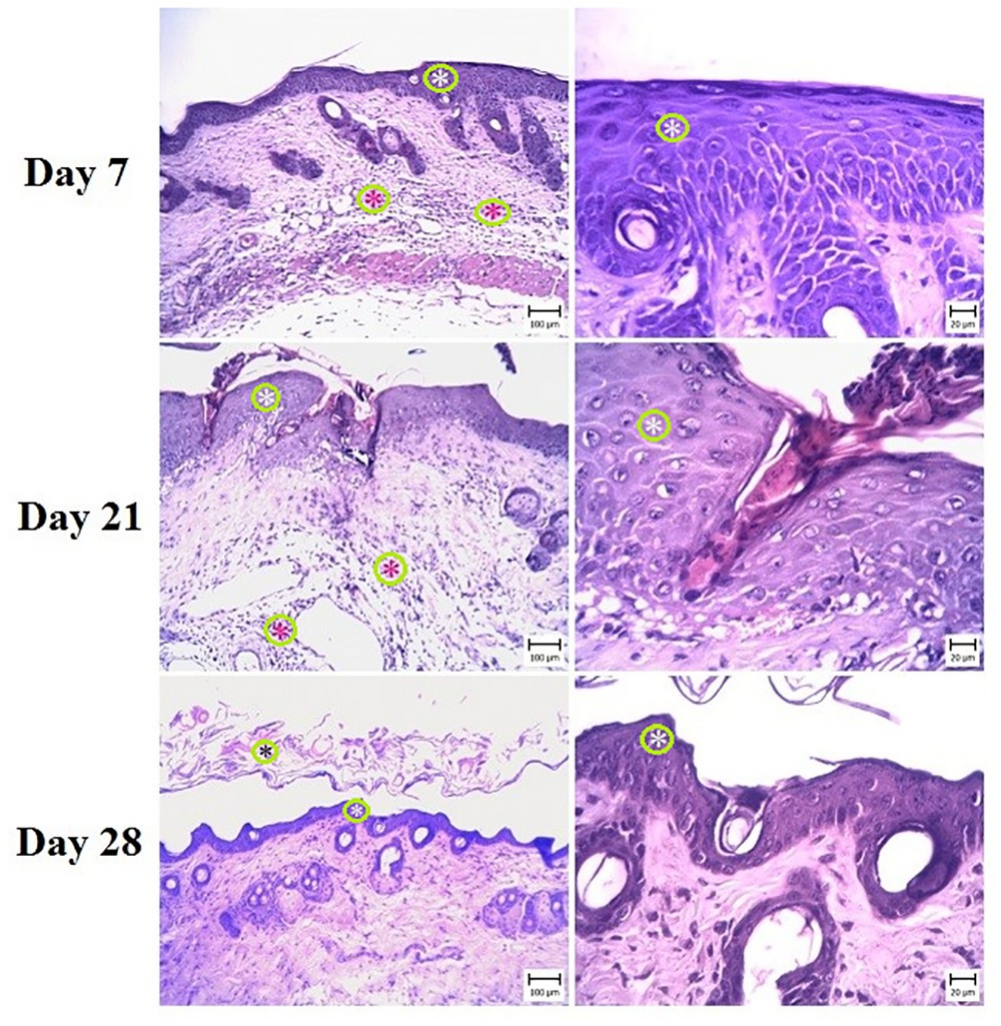
Skin. Mouse in the G6 group (IMQ) (H&E) (Bar = 20–100 μm). Note the acanthosis (white asterisks) (thickening of the stratum spinosum layer) and hyperkeratosis (black asterisks) in the epidermis, which is more severe in day 21. The infiltration of inflammatory cells in the dermis (red asterisks) and dilated capillaries are visible. On day 28, Hyperkeratosis and acanthosis are obvious.

Microscopic evaluation of the skin in G6 (negative control) revealed different lesions, that were like psoriasis symptoms (Fig 1). The thickness of the epidermis was increased due to hyperplasia of keratinocytes (Acanthosis). Hyperkeratosis of epidermal keratinocytes was also seen and there were multiple keratin layers of the epidermis. The dermal cell population, especially the number of mononuclear cells, was increased. A number of these cells were perivascular and a small amount was between the collagen fibrils. The capillaries of the upper and middle portions of the dermis were dilated (Fig 2).

**Fig 3:**
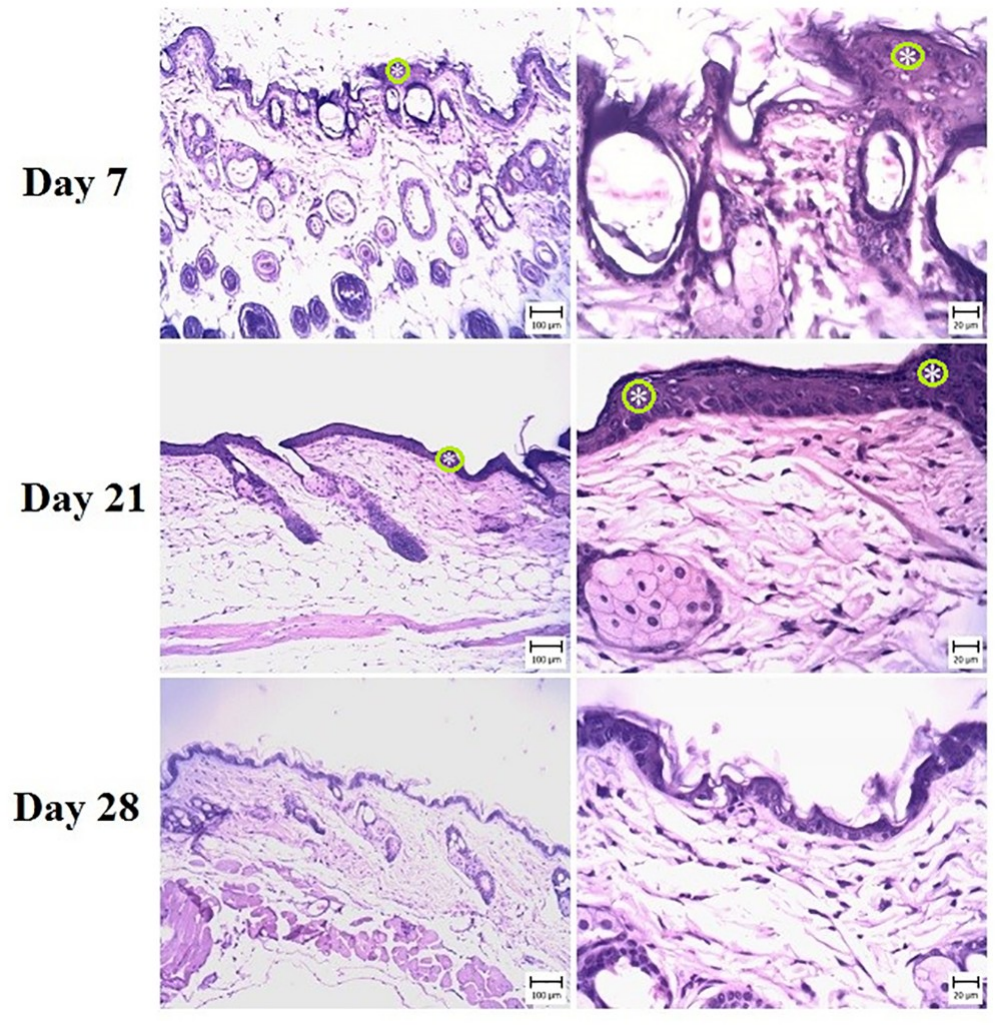
Skin. Mouse in the G2 group (Low dose) (H&E) (Bar = 20–100 μm). Note the acanthosis (white asterisks) (thickening of the stratum spinosum layer) in the epidermis, which is more severe in day 21.

**Fig 4:**
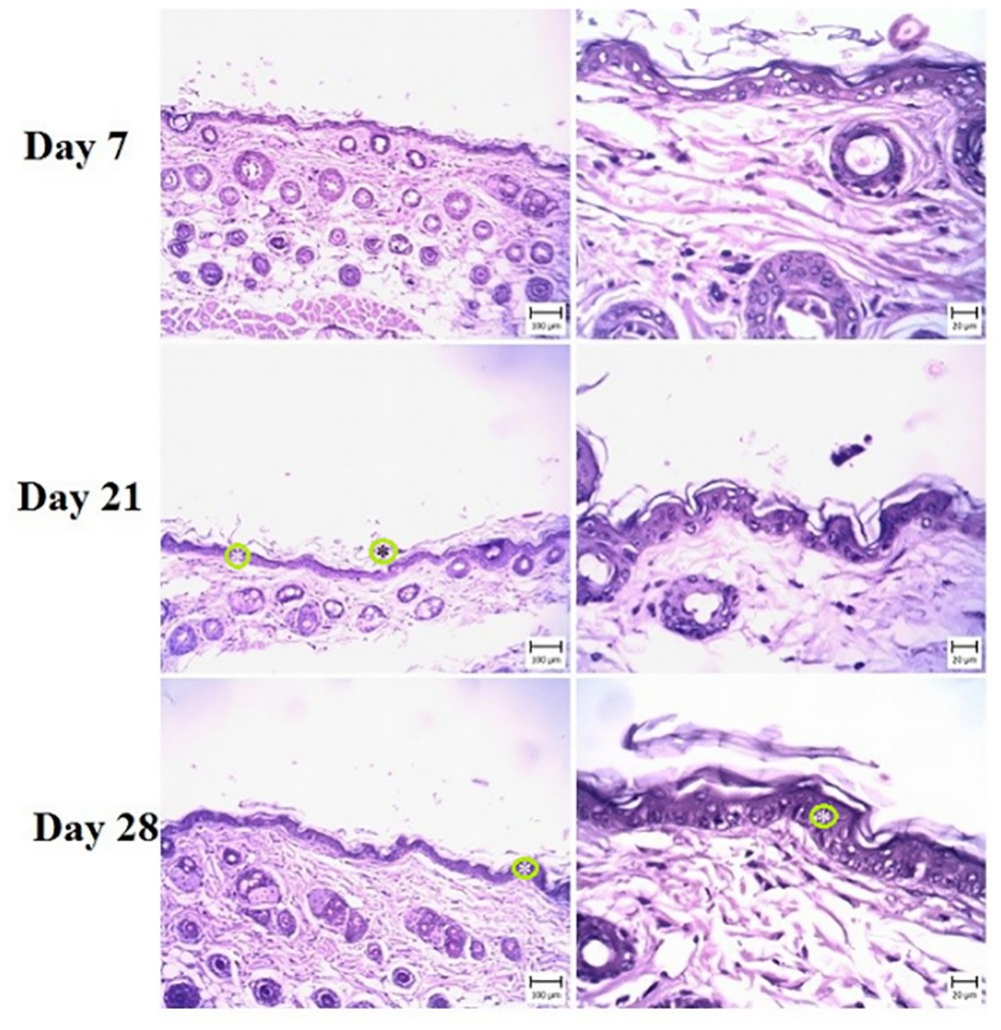
Skin. Mouse in the G3 group (medium dose) (H&E) (Bar = 20–100 μm). Note the acanthosis (white asterisks) (thickening of the stratum spinosum layer) and hyperkeratosis (black asterisks) in the epidermis, which is more severe in day 21 and is less than G2.

**Fig 5:**
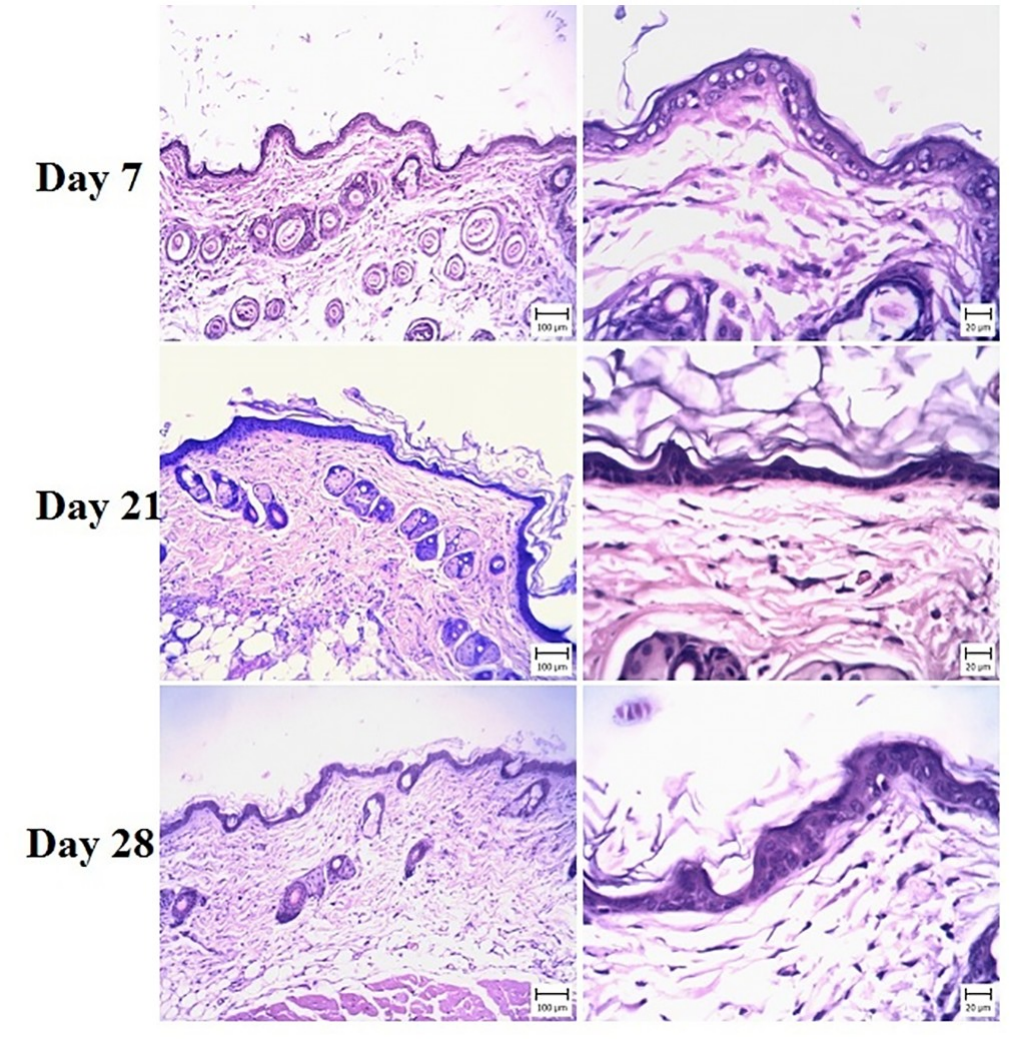
Skin. Mouse in the G4 group (high dose) (H&E) (Bar = 20–100 μm). Note the acanthosis (white asterisks) (thickening of the stratum spinosum layer) and hyperkeratosis (black asterisks) in the epidermis, which is more severe in day 21 and is less than G2 and 3.

**Fig 6:**
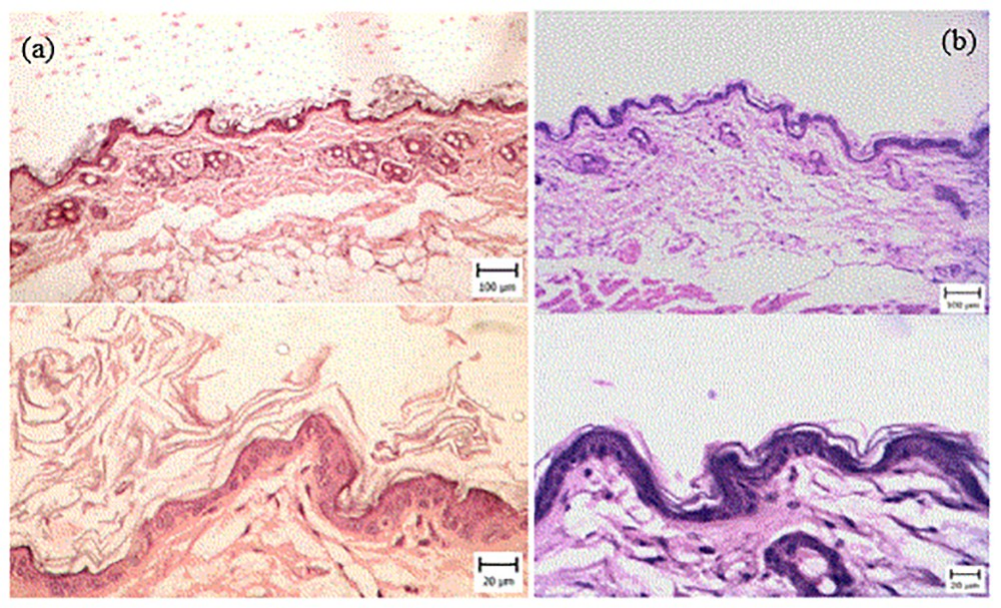
(a) Skin. Mouse in the G5 group (betamethasone) (H&E) (Bar = 20–100 μm). Note the hyperkeratosis and acanthosis in the epidermis after 21 days of treatment, which is decreased in comparison with negative control; (b) Skin. Mouse in the control group (H&E) (Bar = 20–100 μm). Note the normal structure of the epidermis and dermis.

These signs indicated psoriasis lesions after IMQ treatment. In treatment groups, varying degrees of epidermal thickening were detected, which were completely dose-dependent (Figs 3–5). The microscopic view and dermal changes in G2 were the same as in positive control (BMT treated, Fig 6a). This means the efficacy of 200 mg/kg/day methanolic extract in the psoriasis treatment was the same as in positive control. The efficacy of methanolic extract was dose-dependent, and the highest psoriasis treatment was provided by 600 mg/kg/day. In G4 treated by 600 mg/kg/day, no sign of psoriasis lesions was found. On day 28, the severity of lesions did not decrease in G6 (Fig 2). The G1 showed normal skin structure, which was characterized by narrow epidermis with one or two layers of keratinocytes and a slim layer of keratin (Fig 6b). In the dermis, no hyperemia or infiltration of inflammatory cells was seen.

### 3.3. Effect of methanolic extract on the Levels of TNF-α, IL-22, and IL-17A

**Fig 7:**
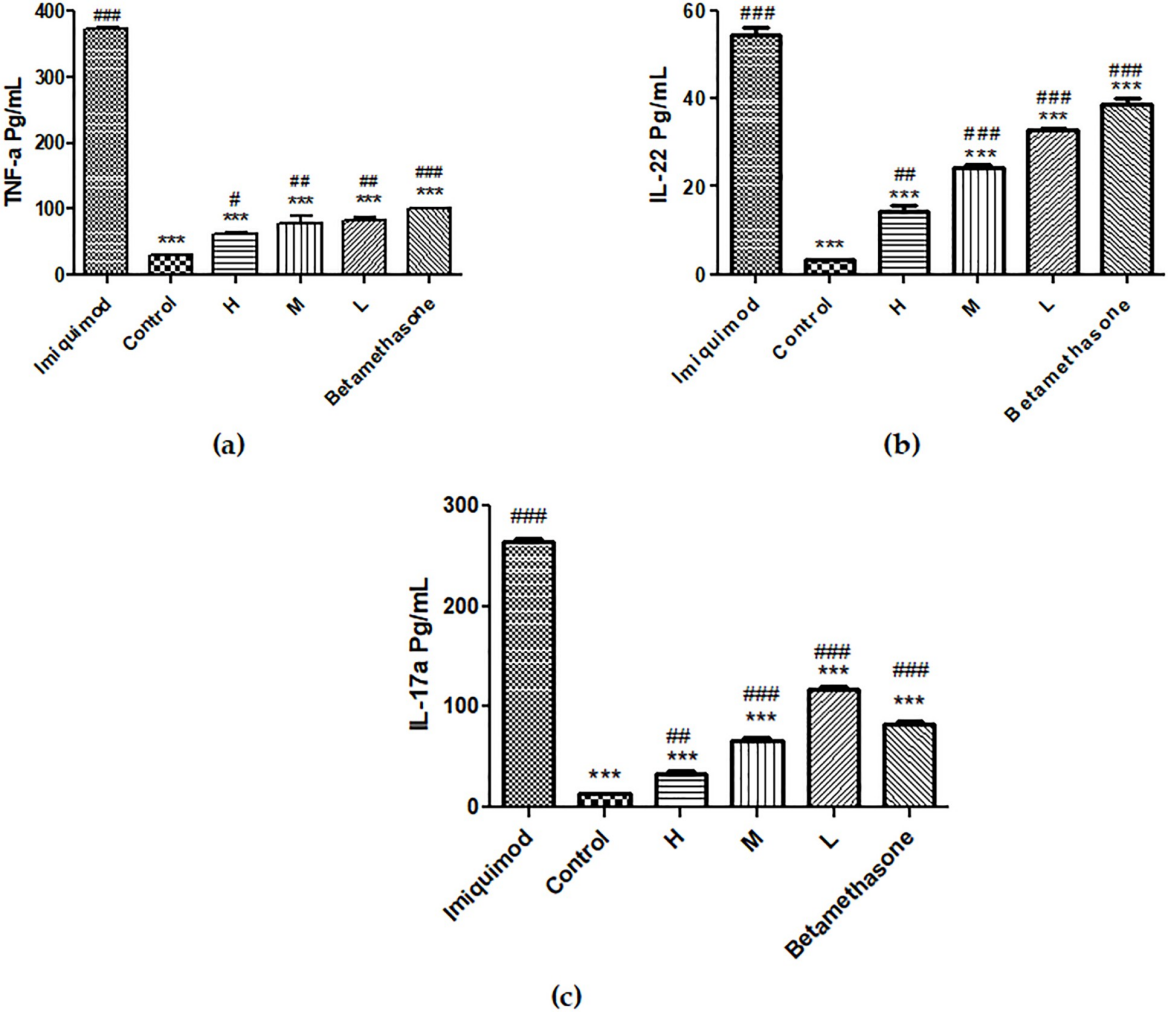
Protein levels of TNF-α, IL-22, and IL-17A in the back skin: (a) TNF-α protein level. Amounts are shown as mean ± SD (*n* = 3 for each group); (b) IL-22 protein levels. Amounts are shown as mean ± SD (*n* = 3 for each group)., (c) IL-17A protein levels. Column with * (in comparison to Imiquimod group) or # (in comparison to control group) above manifests a significant difference between groups (*p*< 0.05). *** *p*< 0.001, # *p*< 0.05, ## *p*< 0.01, ### *p*< 0.001. (G1 group (control), G2 group (L: low dose), G3 group (M: medium dose), G4 group (H: high dose), G5 group (betamethasone), G6 group (IMQ)).

The amounts of IL-22, IL-17A, and TNF-α in different groups are shown in Fig 7. The expression level of TNF-α in G6 (IMQ) was substantially higher than the G1 (normal skin) (371.1 ± 1.626 pg/mL vs. 29.00 ± 1. 697 pg/mL; *p*< 0.001). TNF-α levels in G2-G5 were: 83.55 ± 4.313, 76.95 ± 13.22, 60.50 ± 4.384, and 99.25 ± 0.91 pg/mL. Results indicate that, The level of TNF-a protein expression in skin of G6 (IMQ group) was significantly higher than the G2-G5. The expression level of TNF-α in the back methanolic-extract-treated groups was significantly decreased compared to the G6 (IMQ) in a dose-dependent way (Fig 7a).

In the G6 (IMQ), the expression level of IL-22 was significantly higher than in the G1 (54.55 ± 1.485 pg/mL vs. 3.600 ± 0.323 pg/mL; *p*< 0.001). The level of IL-22 protein expression in skin of G6 was significantly higher than the G2-G5 (32.65 ± 0.63, 24.25 ± 0.63, 14.25 ± 1.485, 38.85 ± 1.344 pg/mL, respectively). It is noteworthy that the level of IL-22 in the back skin of the G4 (H) was not significantly different from that in the G1 (Fig 7b), and was 75% of G6. *Andrographis nallamalayana*, which has been introduced as a potent phytomedicine against psoriasis, reduced the level of IL-22 by less than 70% compared with the IMQ-treated control [21]. The expression levels of IL-22 in the back skin of all G2–G4 groups (low, medium, and high dose) were significantly lower than the G6 (IMQ), in a dose-dependent manner. Interestingly, medium and high doses in G3–G4, with comparatively low levels of IL-22 and TNF-α, showed stronger anti-psoriasis-like symptoms. The expression level of IL-17A in skin of G6 (IMQ) was 261.7 ± 2.828 pg/mL. IL-17A levels in G2-G5 were 117.8 ± 3.845, 65.90 ± 3.536, 33.40 ± 2.121, and 82.40 ± 3.251 pg/mL. Results indicate The level of IL-17A protein expression in the skin of G6 was significantly higher than the G2-G5. The expression level of IL-17A in the back methanolic-extract-treated groups was significantly decreased compared to the G6 (IMQ) in a dose-dependent way (Fig 7c).

### 3.4. Identification of bioactive compounds

**Fig 8:**
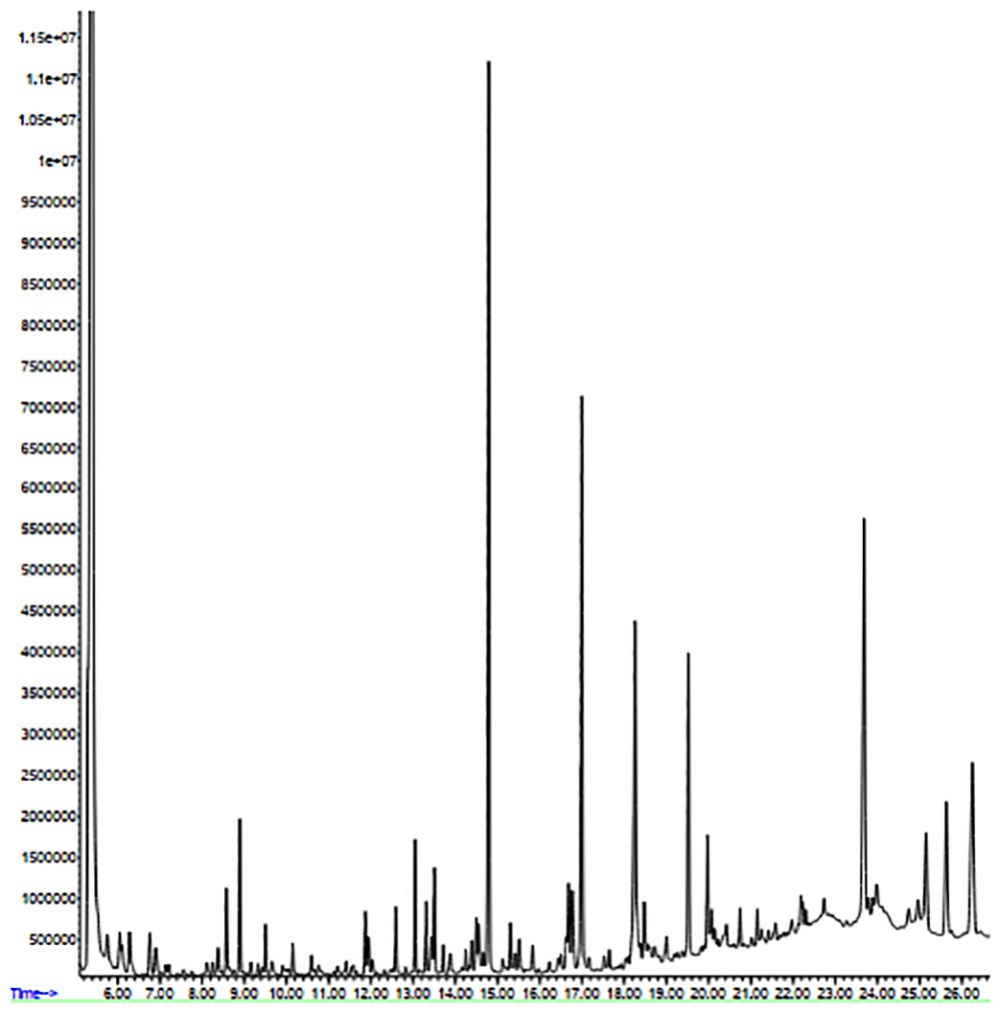
GC-MS chromatogram from methanolic extract of the *Dysidea avara*.

68 chemical compounds were identified in the marine sponge extract using GC-MS (Fig 8 and S1 Table). They include 22-methylcholesta-4,22-dien-3-ol (2.63), cholan-24-oic acid, 3,12-dioxo-,(5.beta.) (0.581), pyrimidine-2,4-diol (0.29), Tetradecanoic acid (0.53), 9,12-octadecadienoic acid (0.26), azelaic acid (0.14), hexadecanoic acid (10.05), oleic acid (1.24), 5-hydroxy-1H-indole-2-carboxylic acid (0.52), 8-Chloro-benzimidazo (2,1-b)quinazoline-12(5H)-one (0.10), etc. The SEA SEARCH SERVER predicted the potential targets of the 68 compounds, determined by GC-MS, to further identify the active compounds of marine sponge extract as anti-inflammatory [31]. These results obtained from The SEA SEARCH SERVER revealed that the 33 compounds presented in Table 2 with anti-inflammatory properties could be the potential active compounds of the marine sponge methanolic extract.

**Table 2:** Active components in *Dysidea avara* extract and their predicted targets.

Number	Compound	Predicted Targets	R.t (min)	Area %
**1**	1-Pentanone, 1-Phenyl	Akr1b1, ALOX15, Alox5, CHRM1, PPARA, PPARG, PLA2G1B, ESR1, Sqle	6.2809	0.5338
**2**	Benzenepropanoic acid	AKR1B1, ALOX15, ALOX5, CXCL8, Egfr, SELE, PLA2G1B, PLA2G2A, Parp2, CYSLTR1, CYSLTR2, Cxcr4, LTA4H, MMP14, Egfr	7.035	0.0509
**3**	8-Chloro-Benzimidazo(2,1-b)Quinazolin-12(5H)-one	PARP1, ADORA1, VGFR2, AKR1B1,	7.145	0.1003
**4**	(*E*)-*N*-(2-Methylpropyl)-3-(-Terthien-5-yl)Propenamide	Alox5,TNFRSF1A	7.2157	0.1117
**5**	Decanoic acid	HMGCR, Alox5, Alox15, PPARG, PPARD, PPARA, PLA2G1B, PLA2G2A, Prkca, Sqle	7.5691	0.0931
**6**	Benzoic acid, 4-Methoxy	AKR1B1, ALOX15, ALOX5,SELE, GSK3B,CHRM1, PPARG, PPARA,PPARD, PLA2G2A, RELA, TNFRSF1A, ADORA2A, Syk, Prkca, CYSLTR1, NtBBF1.1, LTA4H, MMP14,	7.8834	0.1312
**7**	Butyric acid, 4-Phenyl	AKR1B1, ALOX5, ALOX15, CXCL8, Egfr, PPARD, PPARG, PPARA, PLA2G2A, CXCL8, CYSLTR2, CYSLTR1, Cxcr4, LTA4H, MMP14, Egfr, PLA2G2A,	8.5825	0.6755
**8**	Dodecanoic acid	HMGCR, Alox5, Alox15, PPARG, PPARD, PPARA, PLA2G1B, PLA2G2A, Prkca, Sqle	10.1457	0.2275
**9**	Octanedioic acid, bis Ester	HMGCR, Pla2g2a	10.7663	0.1338
**10**	Nonanoic acid	HMGCR, Alox5, Alox15, PPARG, PPARD, PPARA, PLA2G1B, PLA2G2A, Prkca, Sqle	11.3711	0.0411
**11**	Tetradecanoic acid	HMGCR, Alox5, Alox15, PPARG, PPARD, PPARA, PLA2G1B, PLA2G2A, Prkca, Sqle	12.1488	0.537
**12**	1H-Indole-3-Carboxaldehyde	ALOX15, TNFRSF1A, PARP1	12.2431	0.0115
**13**	1H-Indole-2-Carboxylic acid, 5-oxy	GSK3-beta, NGFR, FPR2, Syk, NtBBF1.1	13.3114	0.5294
**14**	n-Pentadecanoic acid	HMGCR, Alox5, Alox15, PPARG, PPARD, PPARA, PLA2G1B, PLA2G2A, Prkca, Sqle	13.7199	0.2743
**15**	Hexadecanoic acid	HMGCR, ALOX15, Alox5, Sqle, PPARA, PPARG, PPARD, PLA2G1B, Prkca, PLA2G2A	14.796	10.0532
**16**	Heptadecanoic acid	HMGCR, Alox5, Alox15, PPARG, PPARD, PPARA, PLA2G1B, PLA2G2A, Prkca, Sqle	15.5187	0.7598
**17**	9,12-Octadecadienoic acid (z,z)	HMGCR, ALOX5, ALOX15, PPARG, PPARA, PPARD, PLA2G1B, PRKCA, CYSLTR1, PLA2G2A, Sqle	16.6263	0.2613
**18**	Oleic acid	HMGCR, ALOX5, ALOX15, PPARG, PPARA, PPARD, PLA2G1B, PRKCA, CYSLTR1, PLA2G2A, CYSLTR1	16.6892	1.2469
**19**	Azonino [5,4-b]Indole-3(2h)-Carboximidic acid, 7-(Aminocarbonyl)-1,4,5,6,7,8-Hexahydro-, Methyl ester	IL2RA, BCHE, MAPK8, STAT3, JUN,	16.7756	0.9277
**20**	Octadecanoic acid	HMGCR, Pla2g2a	16.9955	4.9197
**21**	Nonadecanoic acid	HMGCR, Alox5, Alox15, PPARG, PPARD, PPARA, PLA2G1B, PLA2G2A, Prkca, Sqle	18.0324	0.0798
**22**	9-Octadecenamide, (z)	HMGCR, ALOX5, ALOX15, PPARG, PPARA, PLA2G1B, PRKCA, PLA2G2A, Sqle	18.2602	4.2447
**23**	Octadecanamide	HMGCR, PPARG, PLA2G2A, PLA2G1B, Prkca, Sqle	18.4802	0.6313
**24**	1-o-Heptadecylglycerol	PLA2G1B, PRKCA, Sqle	18.6137	0.1914
**25**	11-Eicosaenoic acid	HMGCR, ALOX5, ALOX15, PPARG, PPARA, PPARD, PLA2G1B, PRKCA, CYSLTR1, PLA2G2A	18.7237	0.3187
**26**	Arachidic acid	HMGCR, ALOX5, ALOX15, PPARA, PPARD, PPARG, PLA2G1B, PRKCA, PLA2G2A, Sqle	19.0065	0.3633
**27**	5,8,11,14,17-Eicosapentaenoic acid, Methyl ester	ALOX15, ALOX5, PRKCA,	20.0748	0.5744
**28**	Docosanoic acid	HMGCR, Alox5, Alox15, PPARG, PPARD, PPARA, PLA2G1B, PLA2G2A, Prkca, Sqle	20.7504	0.5679
**29**	Acetic acid, Mercapto-, Dodecyl ester	PLA2G1B, PLA2G2A, Sqle	21.9758	0.7595
**30**	Cholan-24-oic acid, 3,12-dioxo-, (5.beta.)	CD4	22.6749	0.8129
**31**	Pyrrolidine, 1-(1,6-Dioxooctadecyl)	IL6ST, PLA2G2A, Prkca	23.0912	0.6458
**32**	1-Methylnonyl oxy	Pla2g2a, PLA2G1B	23.6882	5.4093
**33**	22-Methylcholesta-4,22-dien-3-ol	CD4, Esr1	26.2569	2.6304

### 3.5. Method validation for analysis of cholan

#### 3.5.1. Specificity

**Fig 9:**
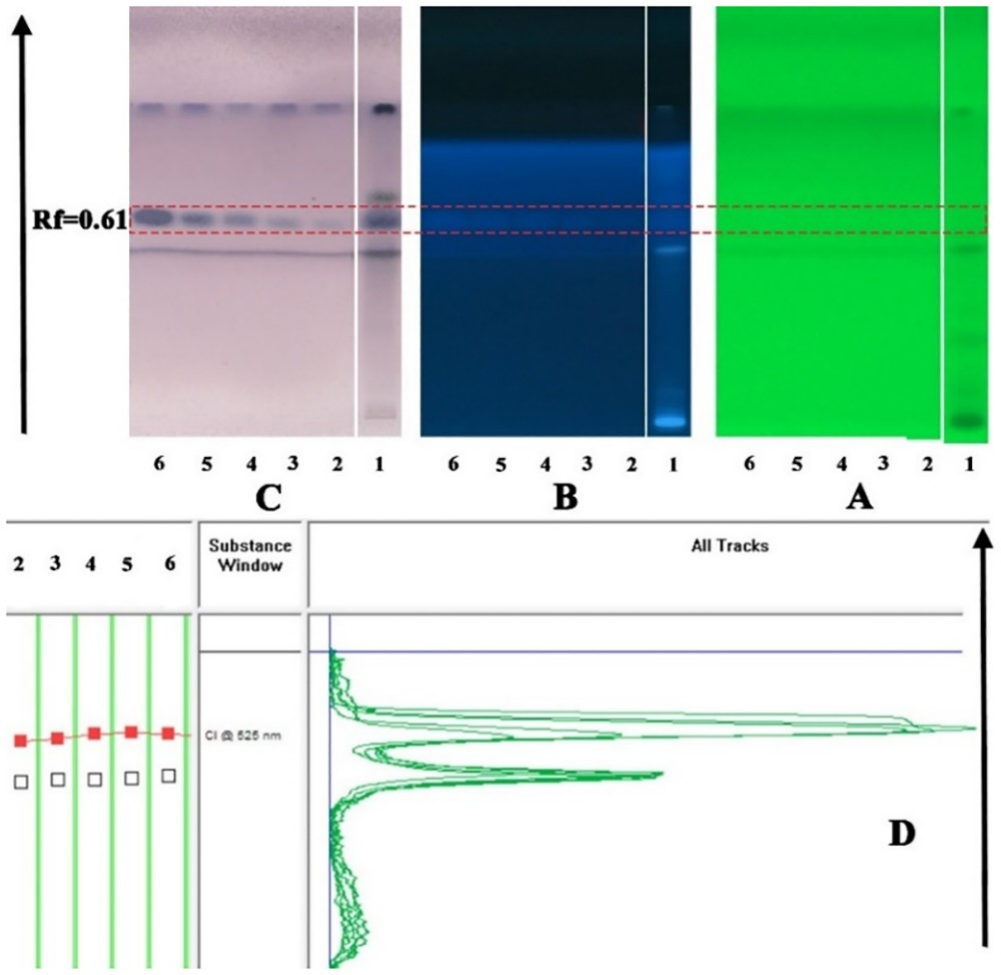
Chromatogram of standard cholan using mobile phase toluene: hexane: methanol: ethyl acetate: acetic acid (60:15:17.5:5:2.5).

Cholan was selected as a major compound in the methanolic extract for skin permeation study. For this purpose, a valid HPTLC technique was needed. The choice of a mobile phase was performed on the grounds of polarity to create the HPTLC technique for the assessment of cholan. A solvent system with a suitable and considerably distinct Rf value for cholan was required that would offer thick and compact spots. The solvent system consisting of toluene: hexane: methanol: ethyl acetate: Acetic acid (60:15:17.5:5:2.5) separated cholan from its methanol extract. This chromatographic system generated a well-defined cholan compact place with an optimal *Rf* = 0.61 (Fig 9). It also provided a decent analyte resolution from other compounds.

#### 3.5.2. Curves in linearity and calibration

Linearity is the capacity of the analytical method to achieve test outcomes within a specified range that is directly proportional to the analyte concentration or through mathematics conversion. The technique was discovered to be linear in a concentration range of 20–400 ug/mL (*n* = 5). The information of regression (Table 3) showed a strong linear connection across the spectrum of the concentration studied, proving its analytical suitability. In standard curve slopes, there were no significant differences (*R*2 = 0.9799).

**Table 3:** Calibration, linearity, accuracy, and precision for cholan determination.

Equations	*R*	Linear range (μg)	LOD (μg)	LOQ (μg)
*y* = 6.5296*x* + 321.24	0.9799	20–400	2.0717	6.9058

LOD: Limit of determination

LOQ: Limit of quantification

#### 3.5.3. Sensitivity

The sensitivity of the method was determined under the experimental conditions employed by calculating the limit of detection (LOD) and limit of quantification (LOQ). LOD and LOQ for cholan-24-oic acid, 3, 12-dioxo-, (5.beta.) were found to be 2.07 and 6.90 ng/band, with RSD < 5% (Table 3).

### 3.6. *In vitro* permeation studies

**Fig 10:**
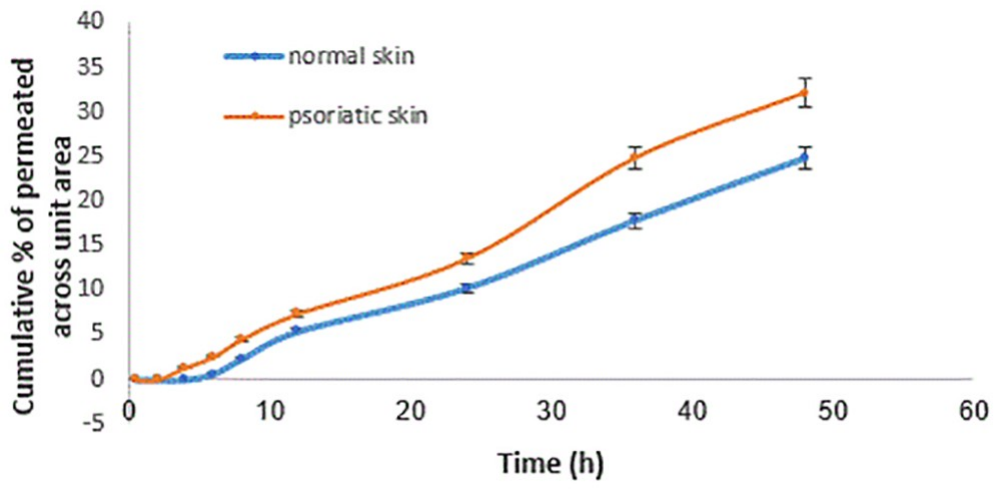
Percentage of cholan permeated through normal and psoriatic skin at different times.

According to a report about the anti-inflammatory effect of the cholan in the literature [32], the HPTLC technique was used to explore the cholan permeation profiles. This compound was selected as a major fragment of *Dysidea avara*. The permeation profiles of this compound through normal and psoriatic skins are shown in Fig 10, and permeability parameters presented in Table 4. Psoriatic demonstrated a more permeable membrane than normal skin, which also has been reported in the previous study [33]. The decrease in skin cellular integration in psoriasis plaque is the main reason for its permeability property. Hydrophilic molecules revealed more permeability through the skin after IMQ treatment. It has been reported that IMQ increased the skin deposition and flux of 5-aminolevulinic acid by 5.6 to 14.4 fold, respectively, and follicular accumulation was also increased by 3.8 fold [34]. The permeability of *Dysidea avara* active components through normal and psoriatic skins has not been reported. Results in the present study showed that more than 2.5 mg cholan (provided by Jss × 48 h) passed through psoriatic skin, which can be reduced by using suitable novel delivery systems such as hydrogels or liposomes. Jss and Q48, defined permeation rate through the skin in steady-state and drug amount permeated after 48 h, respectively.

**Table 4:** Permeation parameters of cholan through normal and psoriatic skins (Mean ± SD, *n* = 5).

Skin type	Jss (mg/cm2. h)	Q48 (mg)	P (cm/h)
Normal	0.043 ± 0.005	1.98 ± 0.2	0.0053 ± 0.0007
Psoriatic	0.059 ± 0.007	2.57 ± 0.21	0.0073 ± 0.0005

Jss: steady-state flux; Q48: cumulative permeated amount after 48 h; P: permeability coefficient

## 4. Discussion

*Dysidea avara* in this study conducted on a mouse model of psoriasis displayed encouraging anti-psoriatic properties. Comparison of IMQ-induced mice psoriasis skin lesion RNA microarray data with psoriasis patients, confirming similar gene expression alterations [35]. This study has several strengths, but also some limitations related to the study type (pilot study) and needs further validation with an omits approach. In this study, the anti-psoriatic activity of *Dysidea avara*'s on the psoriatic mouse model was studied. PASI scoring was used as a quantitative index that reflects the severity of the psoriasis lesions. To date, practical and useful biomarkers for monitoring psoriasis are still missing, furthermore, only the PASI score is used to monitor therapeutic efficacy and it is a reductive approach since psoriasis is a well-established systemic disease. The results showed a dose-dependent treatment trend compared to negative (G6) and positive control (G5), which could substantially improve the severity of psoriatic lesions. Results from pathological sections showed that, in comparison to the normal skin (G1), dyskeratosis, subcutaneous hemorrhage, and inflammatory cell infiltration were shown in G6 (IMQ). Neutrophil infiltration into the epidermis has been shown in histopathological pictures. In psoriasis, neutrophils infiltrate into the epidermis and secrete IL-17A, which induces basal keratinocytes proliferation, followed by the production of IL-23 and TLR4 [36]. Neutrophils infiltration, besides the high value of the IL-17A in the G6 group, indicates the role of IL-17A, IL-23/Th 17 axis in the pathogenesis of psoriasis that is mediated by neutrophils” infiltration. The methanolic extract may inhibit these symptoms with variable degrees of severity. Groups that have been treated with *Dysidea avara* methanolic extracts are most active in reducing symptoms of induced psoriasis by decreasing amounts of IL-22, IL-17A, TNF-α, and decreasing neutrophils” infiltration into the epidermis, which is important in psoriasis’s pathogenesis. We identified 68 compounds from the methanolic extract by GC-MS, and the SEA SEARCH SERVER predicted the anti-inflammatory compounds. Among these compounds, 33 of them could have an anti-inflammatory potential that could treat psoriasis synergistically through different targets and various pathways. The presence of bioactive compounds like tetradecanoic acid, Hexadecanoic acid, and 22-methylcholesta-4, 22-dien-3-ol, and cholan-24-oic acid, 3, 12-dioxo-, (5.beta.) indicates its potential for the development of anti-psoriatic natural products. Several unmet needs that the existent drugs are not addressing may be a fertile territory, which new therapies may eradicate, such as itch-psoriasis-related issues. Future studies should compare anti-psoriatic drugs by the use of biomarkers present in the literature. In overall, Marine sponge *Dysidea avara* can be introduced as potential psoriasis topical treatment if more information including safety and suitable delivery systems obtained in future research.

## Supporting information

S1 File (RAR)

S1 Graphical abstract (TIF)

S1 Fig (DOCX)

S1 Table Chemical constituents of Dysidea avara methanolic extract, detected by GC-MS. (DOCX)

S2 Table Potential targets anti-inflammatory. (DOCX)

S3 Table The peak area data for the calibration curves (*n* = 5). (DOCX)
