# Supplementary figures and images for: Chemical profiling and anti-psoriatic activity of marine sponge (Dysidea avara) in induced imiquimod-psoriasis-skin model
Source: PLoS One. 2020 Nov 30;15(11):e0241582. doi: 10.1371/journal.pone.0241582 (PMC7703918; doi:10.1371/journal.pone.0241582)

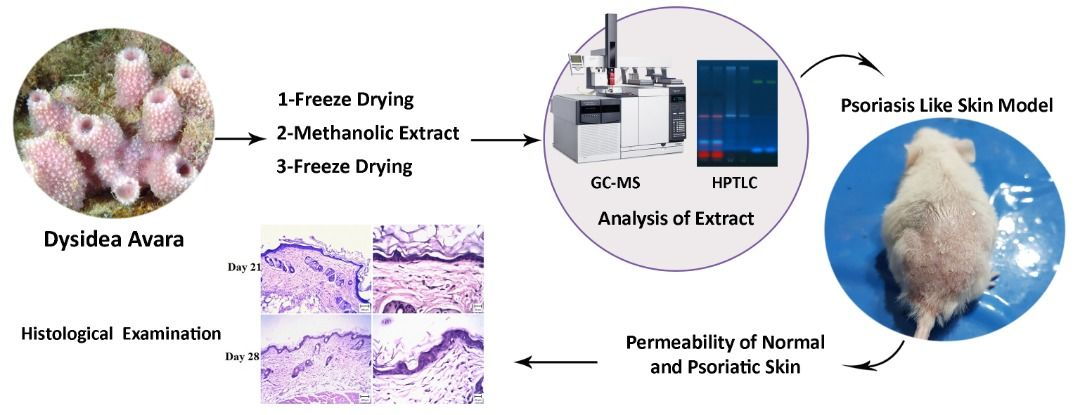

Supplement: S1 Graphical abstract — (TIF) [file pone.0241582.s002.tif]
