# Supplementary material for: Chemical profiling and anti-psoriatic activity of marine sponge (Dysidea avara) in induced imiquimod-psoriasis-skin model
Source: PLoS One. 2020 Nov 30;15(11):e0241582. doi: 10.1371/journal.pone.0241582 (PMC7703918; doi:10.1371/journal.pone.0241582)
Supplement: S1 Fig — (DOCX) [file pone.0241582.s003.docx]

S1 Fig. Linear Regression Data for the Calibration Curves (n=5)
