# Supplementary material for: Chemical profiling and anti-psoriatic activity of marine sponge (Dysidea avara) in induced imiquimod-psoriasis-skin model
Source: PLoS One. 2020 Nov 30;15(11):e0241582. doi: 10.1371/journal.pone.0241582 (PMC7703918; doi:10.1371/journal.pone.0241582)
Supplement: S1 Table — (DOCX) [file pone.0241582.s004.docx]

S1 Table. Chemical constituents of *Dysidea avara* methanolic extract, detected by GC-MS.

| PK | Ret time | Compound name | Area % |
| --- | --- | --- | --- |
| 1 | 5.37 | Silanol, trimethyl-, phosphate | 24.4039 |
| 2 | 6.1081 | pyrimidine-2,4-diol | 0.2937 |
| 3 | 6.2809 | 1-Pentanone, 1-phenyl | 0.5338 |
| 4 | 6.438 | pentadecanoic acid | 0.0119 |
| 5 | 6.5951 | 1,3-Isobenzofurandione, 4-nitro | 0.0233 |
| 6 | 7.035 | Benzenepropanoic acid | 0.0509 |
| 7 | 7.145 | 8-Chloro-benzimidazo(2,1-b)quinazolin-12(5H)-one | 0.1003 |
| 8 | 7.2157 | (E)-N-(2-methylpropyl)-3-(2,2': 5',2''-terthien-5-yl)propenamide | 0.1117 |
| 9 | 7.6556 | Piperidine, 1,1'-methylenebis | 0.0197 |
| 10 | 7.7577 | 2,3-Dimethylthieno[3,2-b]quinolin-4-one | 0.055 |
| 11 | 7.8284 | 1-allyl-4-(methylperoxy)benzene | 0.0147 |
| 12 | 7.8834 | 4-methoxybenzoic acid | 0.1312 |
| 13 | 7.9383 | 4-hydroxybutanoic acid | 0.0236 |
| 14 | 8.1112 | 1,2-Benzenediol, 3,5-di(1,1-dimethylethyl) | 0.1588 |
| 15 | 8.2526 | 4-Chlorobenzenesulphinic acid | 0.1209 |
| 16 | 8.4725 | Cyclohexanecarboxylic acid | 0.0451 |
| 17 | 8.5825 | Butyric acid, 4-phenyl | 0.6755 |
| 18 | 8.6532 | (S)-5-oxopyrrolidine-2-carboxylic acid | 0.0845 |
| 19 | 8.826 | 2-Methyl-3-ethyl-3-hydroxyglutaric acid | 0.0153 |
| 20 | 9.0538 | butyrylglycine | 0.0411 |
| 21 | 9.3287 | 2-Ethyl-3,4-dihydronaphthalene | 0.0935 |
| 22 | 10.1457 | Dodecanoic acid | 0.2275 |
| 23 | 10.3107 | Indole, 3-(2-aminopropyl)-6-fluoro | 0.0167 |
| 24 | 10.3813 | maleic acid | 0.026 |
| 25 | 10.7663 | octanedioic acid | 0.1338 |
| 26 | 10.8684 | Naphtho[1,2-b]furan, 2,3-dihydro-2-(1-methylethenyl) | 0.0333 |
| 27 | 11.214 | glycerol | 0.105 |
| 28 | 11.3711 | Nonanoic acid | 0.0411 |
| 29 | 12.031 | Azelaic acid | 0.1435 |
| 30 | 12.1488 | Tetradecanoic acid | 0.537 |
| 31 | 12.2431 | 1H-indole-3-carbaldehyde | 0.0115 |
| 32 | 12.9108 | 14B-Pregnane | 0.0634 |
| 33 | 13.3114 | 5-hydroxy-1H-indole-2-carboxylic acid | 0.5294 |
| 34 | 13.3114 | 7,9-di-tert-butyl-1-oxaspiro[4.5]deca-6,9-diene-2,8-dione | 0.5294 |
| 35 | 13.7199 | n-pentadecanoic acid | 0.2533 |
| 36 | 14.796 | hexadecanoic acid | 10.0532 |
| 37 | 15.3066 | 1,3-oxathiolo[4,5-b]pyridine, 2,5-dimethyl | 0.3963 |
| 38 | 15.5187 | heptadecanoic acid | 0.7598 |
| 39 | 16.6263 | (9Z,12Z)-octadeca-9,12-dienoic acid | 0.2613 |
| 40 | 16.6892 | oleic acid | 1.2469 |
| 41 | 16.7756 | azonino[5,4-b]indole-3(2h)-carboximidic acid, 7-(aminocarbonyl)-1,4,5,6,7,8-hexahydro-, methyl ester | 0.9277 |
| 42 | 16.9955 | octadecanoic acid | 4.9197 |
| 43 | 17.1683 | p-menth-1-en-3-one, semicarbazone | 0.1359 |
| 44 | 17.6475 | 1a,7b-dihydroazirine(5,6)benzo(1,2-c:3,4,-c')dithiophene | 0.1674 |
| 45 | 18.0324 | nonadecanoic acid | 0.0798 |
| 46 | 18.0874 | methyl (2E,6E,10E)-3,7,11,15-tetramethylhexadeca-2,6,10,14-tetraenoate | 0.0981 |
| 47 | 18.2602 | 9-octadecenamide, (z) | 4.2447 |
| 48 | 18.4802 | Octadecanamide | 0.6313 |
| 49 | 18.6137 | 1-o-heptadecylglycerol | 0.1914 |
| 50 | 18.7237 | 11-eicosenoic acid | 0.3187 |
| 51 | 18.8337 | Scillarenin | 0.1138 |
| 52 | 19.0065 | arachidic acid | 0.3633 |
| 53 | 19.2029 | 2,5-dimethyl-1H-indole | 0.2387 |
| 54 | 19.902 | 4-(isopropylthio)-6-(4-methoxyphenyl)-2-methylnicotinonitrile | 0.1466 |
| 55 | 19.9805 | bis(2-ethylhexyl) phthalate | 1.0229 |
| 56 | 20.0748 | 5,8,11,14,17-eicosapentaenoic acid, methyl ester | 0.5744 |
| 57 | 20.1455 | (1aR,7bS)-1a,7b-dihydro-1H-dithieno[3',4':3,4;3'',4'':5,6]benzo[1,2-b]azirine | 0.3261 |
| 58 | 20.7504 | docosanoic acid | 0.5679 |
| 59 | 21.4181 | (1's,6s)-5-(4-nitrobenzoyloxy)-2',2',6',7-tetramethyl-spiro[benzofuran-2(3h),1'-cyclohexane] | 0.311 |
| 60 | 21.9758 | dodecyl 2-mercaptoacetate | 0.7595 |
| 61 | 22.1879 | spiro[androstane-17,2'-oxiran]-3-ol, 3'-(2-pyridinyl)-, acetate(ester), (3.beta.,3's,5.alpha.,17.beta.) | 0.5264 |
| 62 | 22.6749 | cholan-24-oic acid, 3,12-dioxo-, (5.beta.) | 0.8129 |
| 63 | 22.8556 | 6,7-dihydro-5.beta.,6.beta.-dihydroxy-2,3,9,10-tetramethoxy-7.alpha.-(1,2-epoxyethyl)-5h-dibenzo[a,c]cycloheptene | 2.3511 |
| 64 | 23.0912 | pyrrolidine, 1-(1,6-dioxooctadecyl) | 0.6458 |
| 65 | 23.6882 | decan-2-ol | 5.4093 |
| 66 | 25.1572 | (3.beta.,20r)-20-[2-(1-methylethyl)cyclopropyl]pregn-5-en- 3-ol | 1.8028 |
| 67 | 25.6364 | Cholestrol | 1.6496 |
| 68 | 26.2569 | 22-methylcholesta-4,22-dien-3-ol, (trans) | 2.6304 |
