# Supplementary material for: Chemical profiling and anti-psoriatic activity of marine sponge (Dysidea avara) in induced imiquimod-psoriasis-skin model
Source: PLoS One. 2020 Nov 30;15(11):e0241582. doi: 10.1371/journal.pone.0241582 (PMC7703918; doi:10.1371/journal.pone.0241582)
Supplement: S2 Table — (DOCX) [file pone.0241582.s005.docx]

S2 Table. Potential targets anti-inflammatory.

| No. | Protein Name | Gene Name |
| --- | --- | --- |
| P_01 | 3-hydroxy-3-methylglutaryl-Coenzyme A reductase | HMGCR |
| P_02 | Aldose reductase | AKR1B1 |
| P_03 | Arachidonate 15-lipoxygenase | ALOX15 |
| P_04 | Arachidonate 5-lipoxygenase | ALOX5 |
| P_05 | Baculoviral IAP repeat-containing protein 5 | BIRC5 |
| P_06 | C-C motif chemokine 2 | CCL2 |
| P_07 | CD40 ligand | CD40LG |
| P_08 | Cell division protein kinase 2 | CDK2 |
| P_09 | Cholinesterase | BCHE |
| P_10 | C-X-C motif chemokine 10 | CXCL10 |
| P_11 | C-X-C motif chemokine 2 | CXCL2 |
| P_12 | Tumor necrosis factor receptor superfamily member 16 | NGFR |
| P_13 | Epidermal growth factor receptor | EGFR |
| P_14 | E-selectin | SELE |
| P_15 | Glycogen synthase kinase-3 beta | GSK3B |
| P_16 | Heme oxygenase 1 | HMOX1 |
| P_17 | Inhibitor of nuclear factor kappa-B kinase subunit alpha | CHUK |
| P_18 | Inhibitor of nuclear factor kappa-B kinase subunit beta | IKBKB |
| P_19 | Intercellular adhesion molecule 1 | ICAM1 |
| P_20 | Interleukin-1 alpha | IL1A |
| P_21 | Interleukin-1 beta | IL1B |
| P_22 | Interleukin-10 | IL10 |
| P_23 | Interleukin-2 | IL2 |
| P_24 | Interleukin-6 | IL6 |
| P_25 | Interleukin-8 | IL8 |
| P_26 | Mitogen-activated protein kinase 14 | MAPK14 |
| P_27 | Mitogen-activated protein kinase 8 | MAPK8 |
| P_28 | Muscarinic acetylcholine receptor M1 | CHRM1 |
| P_29 | NF-kappa-B inhibitor alpha | NFKBIA |
| P_30 | Nitric-oxide synthase, endothelial | NOS3 |
| P_31 | Ornithine decarboxylase | ODC1 |
| P_32 | Osteopontin | SPP1 |
| P_33 | Peroxisome proliferator-activated receptor alpha | PPARA |
| P_34 | Peroxisome proliferator-activated receptor delta | PPARD |
| P_35 | Peroxisome proliferator-activated receptor gamma | PPARG |
| P_36 | Phospholipase A2 | PLA2G1B |
| P_37 | Poly [ADP-ribose] polymerase 1 | PARP1 |
| P_38 | Prostaglandin G/H synthase 1 | COX1 |
| P_39 | Prostaglandin G/H synthase 2 | COX2 |
| P_40 | Signal transducer and activator of transcription 1-alpha/beta | STAT1 |
| P_41 | Transcription factor AP-1 | JUN |
| P_42 | Transcription factor p65 | RELA |
| P_43 | Tumor necrosis factor | TNF |
| P_44 | Adenosine receptor A1 | ADORA1 |
| P_45 | Adenosine receptor A2a | ADORA2A |
| P_46 | S100 calcium-binding protein A7 | S100A7 |
| P_47 | S100 calcium-binding protein A8 | S100A8 |
| P_48 | Tyrosine-protein kinase HCK | HCK |
| P_49 | C-X-C motif chemokine 1 | CXCL1 |
| P_50 | Heat shock 70 kDa protein 1A | HSPA1A |
| P_51 | Plasminogen activator inhibitor 1 | SERPINE1 |
| P_52 | C-C motif chemokine 19 | CCL19 |
| P_53 | C-C motif chemokine 5 | CCL5 |
| P_54 | C-C motif chemokine 4 | CCL4 |
| P_55 | C-C motif chemokine 22 | CCL22 |
| P_56 | N-formyl peptide receptor 2 | FPR2 |
| P_57 | Glycogen synthase kinase-3 alpha | GSK3A |
| P_58 | Tyrosine-protein kinase Lyn | LYN |
| P_59 | Chymotrypsin-like elastase family member 1 | CELA1 |
| P_60 | Tyrosine-protein kinase SYK | SYK |
| P_61 | Estrogen receptor | ESR1 |
| P_62 | Tyrosine-protein phosphatase non-receptor type 2 | PTPN2 |
| P_63 | Protein kinase C alpha type | PRKCA |
| P_64 | Proto-oncogene tyrosine-protein kinase Src | SRC |
| P_65 | Inosine-5'-monophosphate dehydrogenase 1 | IMPDH1 |
| P_66 | Inosine-5'-monophosphate dehydrogenase 2 | IMPDH2 |
| P_67 | Cysteinyl leukotriene receptor 1 | CYSLTR1 |
| P_68 | Cysteinyl leukotriene receptor 2 | CYSLTR2 |
| P_69 | B-cell receptor CD22 | CD22 |
| P_70 | Integrin alpha L | CD11A |
| P_71 | cluster of differentiation 2 | CD2 |
| P_72 | lymphocyte function-associated antigen-1 | LFA-1 |
| P_73 | lymphocyte function-associated antigen-3 | LFA-3 |
| P_74 | T-cell surface glycoprotein CD4 | CD4 |
| P_75 | Integrin α4β1 | CD49d(VLA-4) |
| P_76 | Zn finger protein | NtBBF1.1 |
| P_77 | Interleukin-8 | CXCL8 |
| P_78 | Interleukin-20 | CXCL20 |
| P_79 | Interferon alpha/beta receptor 2 | IFNAR2 |
| P_80 | Leukotriene A-4 hydrolase | LTA4H |
| P_81 | Tyrosine-protein kinase JAK1 | JAK1 |
| P_82 | Vascular endothelial growth factor A | VEGFA |
| P_83 | Toll-like receptor 7 | TLR7 |
| P_84 | Toll-like receptor 8 | TLR8 |
| P_85 | psoriasis susceptibility 1 | PSORS1 |
| P_86 | psoriasis susceptibility 2 | PSORS2 |
| P_87 | Tyrosine-protein phosphatase non-receptor type 22 | PTPN22 |
| P_88 | Late Cornified Envelope 3B | LCE3B |
| P_89 | Late Cornified Envelope 3C | LCE3C |
| P_90 | Transcription activator-like effector | TALE |
| P_91 | Matrix metalloproteinase-14 | MMP14 |
| P_92 | Metalloproteinase inhibitor 1 | Timp1 |
| P_93 | Interleukin-6 receptor subunit beta |  |
| P_94 | Phospholipase A2, membrane associated | PLA2G2A |
| P_95 | Squalene mono oxygenase | Sqle |
| P_96 | Interleukin-2 receptor subunit alpha | IL2RA |
| P_97 | Signal transducer and activator of transcription 3 |  |
| P_98 | C-X-C chemokine receptor type 4 | Cxcr4 |
