# Supplementary material for: Chemical profiling and anti-psoriatic activity of marine sponge (Dysidea avara) in induced imiquimod-psoriasis-skin model
Source: PLoS One. 2020 Nov 30;15(11):e0241582. doi: 10.1371/journal.pone.0241582 (PMC7703918; doi:10.1371/journal.pone.0241582)
Supplement: S3 Table — (DOCX) [file pone.0241582.s006.docx]

S3 Table. The peak area Data for the Calibration Curves (n=5)

| C (ug/ml) | Area |
| --- | --- |
| 20 | 293 |
| 50 | 761 |
| 100 | 920 |
| 200 | 1812 |
| 400 | 2848 |
